# AMPK - Activated Protein Kinase and its Role in Energy Metabolism of the Heart

**DOI:** 10.2174/157340310793566073

**Published:** 2010-11

**Authors:** Florian Heidrich, Hanna Schotola, Aron F Popov, Christian Sohns, Julia Schuenemann, Martin Friedrich, Kasim O Coskun, Dirk von Lewinski, Jose Hinz, Martin Bauer, Suyog A Mokashi, Samuel Sossalla, Jan D Schmitto

**Affiliations:** 1 Department of Thoracic, Cardiac and Vascular Surgery, University of Goettingen, Goettingen, Germany; 2 Department of Anesthesiology, Emergency and Intensive Care Medicine, University of Goettingen, Goettingen, Germany; 3 Department of Cardiology, University of Goettingen, Goettingen, Germany; 4 Department of Cardiology, University of Graz, Graz, Austria; 5 Division of Cardiac Surgery, Brigham and Women’s Hospital, Harvard Medical School, Boston, MA, USA

**Keywords:** Adenosine monophosphate - activated protein kinase, AMPK, heart failure, cardiac energy metabolism.

## Abstract

Adenosine monophosphate – activated kinase (AMPK) plays a key role in the coordination of the heart’s anabolic and catabolic pathways. It induces a cellular cascade at the center of maintaining energy homeostasis in the cardiomyocytes.. The activated AMPK is a heterotrimeric protein, separated into a catalytic α -  subunit (63kDa), a regulating β - subunit (38kDa) and a γ - subunit (38kDa), which is allosterically adjusted by adenosine triphosphate (ATP) and adenosine monophosphate (AMP). The actual binding of AMP to the γ – subunit is the step which activates AMPK.

AMPK serves also as a protein kinase in several metabolic pathways of the heart, including cellular energy sensoring or cardiovascular protection. The AMPK cascade represents a sensitive system, activated by cellular stresses that deplete ATP and acts as an indicator of intracellular ATP/AMP.  In the context of cellular stressors (i.e. hypoxia, pressure overload, hypertrophy or ATP deficiency) the increasing levels of AMP promote allosteric activation and phosphorylation of AMPK. As the concentration of AMP begins to increase, ATP competitively inhibits further phosphorylation of AMPK. The increase of AMP may also be induced either from an iatrogenic emboli, percutaneous coronary intervention, or from atherosclerotic plaque rupture leading to an ischemia in the microcirculation. To modulate energy metabolism by phosphorylation and dephosphorylation is vital in terms of ATP usage, maintaining transmembrane transporters and preserving membrane potential.

In this article, we review AMPK and its role as an important regulatory enzyme during periods of myocardial stress, regulating energy metabolism, protein synthesis and cardiovascular protection.

## INTRODUCTION

Adenosine monophosphate-activated kinase (AMPK) induces a cellular cascade at the center of maintaining energy homeostasis in the cell. AMPK received attention in 1973 during a survey of the Acetyl-CoA-carboxylase (ACC) enzyme, an integral enzyme in fatty acid synthesis [1]. Carlson and Kim showed that ACC was regulated *via* phosphorylation and dephosphorylation [1, 2]. Specifically, Carlson described AMPK as an enzyme which works as a function of the intracellular ATP/AMP quotient, which regulates ACC in response to energetic stress [1, 2]. 

**Fig. (1):**
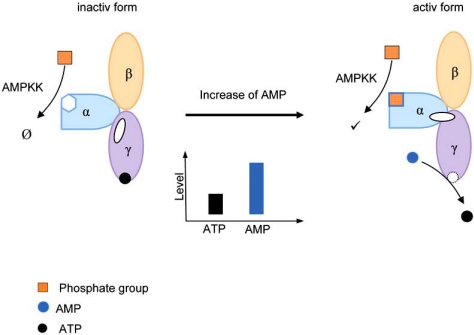
Schematic conformational change of AMPK during alteration of ATP/AMP-quotient enhance threonine172 phosphorylation.

The activated AMPK is a heterotrimeric protein, separated into a catalytic α - subunit (63kDa), a regulating β - subunit (38kDa) and a γ - subunit (38kDa), which is allosterically adjusted by adenosine triphosphate (ATP) and adenosine monophosphate (AMP) [2]. The actual binding of AMP to the γ-subunit activates AMPK. This process occurs by three different pathways: (1) allosteric activation, (2) conformational changes and (3) inhibition of dephosphorylation (Fig. **1**). In the second pathway, a conformational change in the α- subunit by the γ-subunit facilitates the phosphorylation of the α-subunit by an upstream AMPK kinases (AMPKK) e.g. serine/threonine kinase LKB1 and calmodulin-dependent protein kinase kinase (CaMKK) (Fig. **1**). The third pathway, the inhibition of dephosphorylation, can occur by several protein phosphatases [2].

AMPK serves as a protein kinase in several metabolic pathways of the heart, including cellular energy sensoring or cardiovascular protection [3-5] - previously examined in animal models [6-9]. In the context of cellular stressors (i.e. hypoxia, pressure overload, hypertrophy or ATP deficiency) the increasing levels of AMP promote allosteric activation and phosphorylation of AMPK [3-5]. On the other hand, increase of AMP may also be induced either from an iatrogenic emboli, percutaneous coronary intervention, or from atherosclerotic plaque rupture leading to an ischemia in the microcirculation activates AMPK. This competitive inhibition may also be induced either from an iatrogenic emboli, percutaneous coronary intervention, or from atherosclerotic plaque rupture leading to an ischemia in the microcirculation. To modulate energy metabolism by phosphorylation and dephosphorylation is vital in terms of ATP usage, maintaining transmembrane transporters and preserving membrane potential. 

The exact role of AMPK in cardiac metabolism is highly controversial [4]. Calcium concentration plays a pivotal role in the contraction and relaxation of the myocardium. Calcium-dependent ATPases are localized in the endoplasmatic reticulum (ER) and orchestrate the intracellular calcium concentration. As the calcium concentration rises, CaMKK activates AMPK by phosphorylation [10]. Highly regulated transporters prevent calcium unbalance which could lead to a mismatch between myocardial contractility and relaxation. In the acute phase of ischemia, AMPK inhibits anabolic metabolism and ATP–consuming biosynthetic pathways and supports catabolic energy-generating pathways. However, AMPK chronically activated may inhibit overall energy metabolism. This observation was first postulated by Dyck *et al*. and Carvajal *et al*. [4, 11], who reported the effects of AMPK in cardiac metabolism. The contrary effects could be described by the following:

After the first adaption realized e.g. by saving ATP or raising glucose admission, some biosyntheses are strictly needful to preserve the cells life. On the other side the down regulation of AMPK leads to increase injury when the coronary blood flow is curbed. In general we could establish the hypothesis that AMPK works as a key stress signalling enzyme in heart or as a cellular energy sensor for cardiovascular protection [3, 4].

## AMPK AND FATTY ACID METABOLISM

**Fig. (2):**
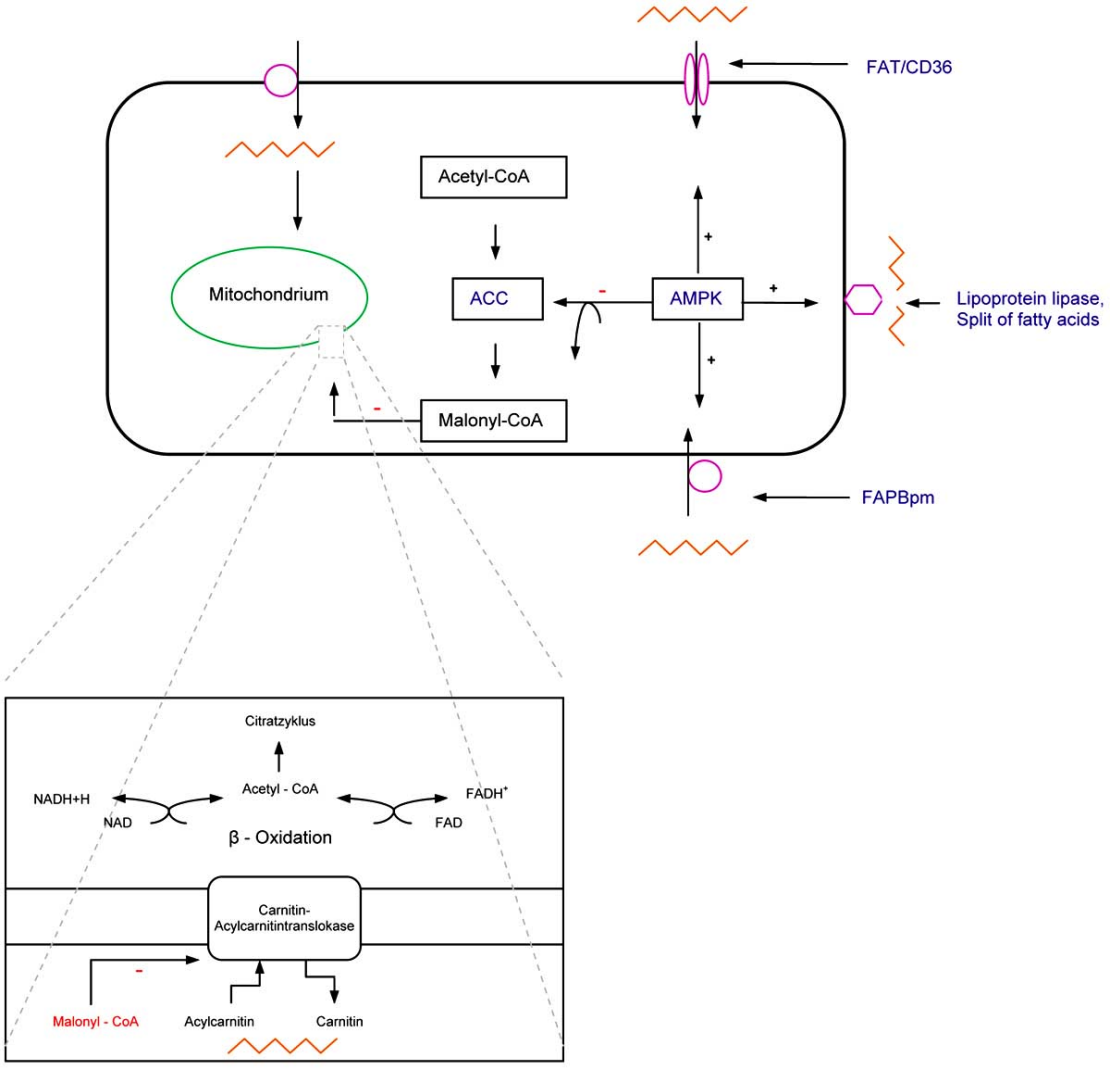
Fat acid metabolism. ACC Acetyl-CoA carboxylaseFAPBpm Fatty acid binding proteinFAT/CD36 Fatty acid transporter (CD36)

The heart uses various substrates for managing the energy metabolism, mainly fatty acids and pyruvate, 60-90 % and 10-40%, respectively [4]. Cardiomyocytes are able to switch between substrates for energy, either the oxidation fatty acids or glucose, depending on the different situations e.g. ischemia or anoxia. Thus, fatty acid metabolism is a significant target for storing or creating energy for the heart. Therefore, AMPK modulates the metabolism of fatty acids in different ways. For example, AMPK phosphorylates the acetyl-CoA carboxylase (ACC) at the amino acid sites Ser79, Ser1200 and Ser1215 [12, 13]. The phosphorylation and inhibition of ACC stimulates the transport of fatty acids into mitochondria, where the beta–oxidation is localized. Processing and consuming of fatty acids by cardiomyocytes requires the carnitine-carrier-system (CCS). The CCS transports acyl-carnitine into the mitochondria and carnitine into the cytosol. The carnitine palmitoyltransferase (CTP–1), which synthesized acyl - carnitin, is regulated by malonyl-CoA, the first product of fat acid biosynthesis (Fig. **2**) [12-14]. Allosteric regulation of CTP-1 by malonyl-CoA enables the biosynthesis of fatty acids. 

Increasing the translocation of the lipoprotein lipase into the membrane subsequently increases the supply of fatty acids (Fig. **2**). It is indispensible to segregate e.g. chylomicrons, triacylglycerides (TAGs), very low density lipoproteins (VLDLs) and low density lipoproteins (LDLs) into a single fatty acid. These individual fatty acids will channel into the beta-oxidation to generate reduced agent for acquire energy [13].

Other targets of energy metabolism are the upstream fatty acid transporter (FAT/CD36) and the membrane associated fatty acid binding protein (FAPBpm), which are responsible for fatty acid uptake e.g. in the heart [3, 4]. Here, AMPK elevates the necessary protein expression to transport free fatty acids into cardiomyocytes (Fig. **2**). The absence of AMPK results in a reduction of oxidation seen in cardiomyocytes, thus cardiomyocytes rely on other substrates such as lipids or pyruvate [15]. Each pathway increases the concentration of NADH/H^+^ and FADH_2_ to extract ATP. NADH/H^+ ^and FADH_2 _work as reducing agents which are necessary to sustain the respiratory chain, located on the inner mitochondrial membrane, with electrons. Generating ATP is essential for cardiomyocyte electrolyte balance and cellular protein volume.

## AMPK AND GLUCOSE METABOLISM

AMPK plays an important role in the metabolism of glucose, producing the majority of ATP, second to the fatty acid oxidation in heart [16]. After AMP is activated, the levels of glucose uptake increase *via* either glucose transporter 4 (GLUT 4) or up regulation of GLUT 1 by AMPK activation [3, 4, 16]. The targets of phosphorylation by AMPK and its mediators are very diverse, e.g. protein kinase C (PKC), p38 mitogen-activated protein kinase or binding protein complex 1. Each target is a central part in various signal transduction pathways, especially PKC and intracellular transports [9, 26]. Glucose transporters are very connotative for glucose absorption and become translocated into cardiomyocyte’s cell membranes. 

**Fig. (3):**
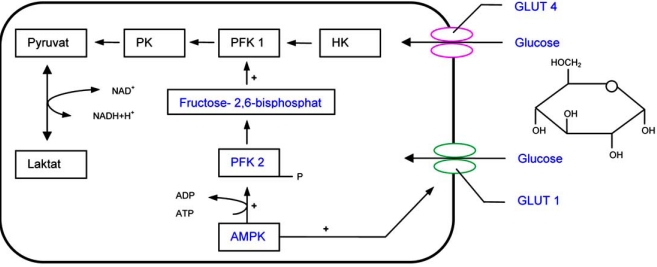
Carbohydrate metabolism HK HexokinasePFK PhospofructokinasePK PyruvatkinaseGLUT Glucosetransporter

AMPK phosphorylates the 6-phosphofructo-2-kinase (PFK 2), a key enzyme of glucose depletion, to activate glycolysis during states of myocardial ischemia, exercise and anoxia [2, 5, 10, 14]. This activation is competitively regulated. Fructose 2, 6 bisphosphate stimulates 6-phospho-fructo-1-kinase (PFK 1), which is a celerity dependent step in glycolysis (Fig. **3**). PFK 1 is an essential regulatory step in the glycolytic pathway. 

The direct phosphorylation of glycogen-synthase and phosphorylase-kinase, each key enzymes of glycogen metabolism, influences glycogen storage. Thus, AMPK induces a more efficient storage and utilization of glucose [16]. It is important to mention that the AMPK-mediated modulation of the above mentioned gene expression targets leads to cardiac protection from anoxia and ischemia. 

## AMPK IN HEART FAILURE

Hemodynamic disorders, for example oxidative stress or heart failure, either benefits, induces or instigates an inflammatory reaction [34]. The migration of macrophages, monocytes and leukocytes and the expression of different proinflammatory cytokines including TNF-α, IL - 1β, IL 6 are responsible for the systemic reactions [17, 18]. 

During an inflammatory reaction the activity of AMPK increases secondary to the release of MIF (macrophage migration inhibitory factor). Therefore MIF, a cytokine influencing multiple aspect of systemic reactions during inflammation, stimulates AMPK through CD74 [19]. In addition, MIF has the ability to modulate glucose uptake in hypoxic conditions. Given that, AMPK can regulate several pathways of energy metabolism in the heart during inflammation. 

AMPK is involved in different pro-apoptotic (p53 protein, BAX, p38 MAPK) or antiapoptotic pathways. During physical or chemical stress, AMPK activation is anti-apoptotic [20]. During an acute lung injury, AMPK abates the pro-inflammatory reaction mediated by neutrophil granulocytes and therefore the extent of functional deficit [17]. During cellular stress such as hypoxia, the protein degradation process plays an important role in managing the intracellular protein volume, another regulation target for AMPK. The increased incidence of autophage during myocardial ischemia shows a correlation with AMPK [21]. 

Several proteins (e.g. eEF-2K) or transcription factors (e.g. p27) stimulate AMPK and impair cardiomyocytes [22, 23]. These metabolic pathways are seen in conditions such as myocardial ischemia and ischemia/reperfusion, demonstrating the important role of AMPK during ischemia compared to apoptosis or necrosis [22]. For example analogous to myocardial metabolism, during acute renal ischemia AMPK levels increase rapidly to orchestrate energy metabolism. The modulation of specific sodium transporters in kidney by AMPK is not well described [24]. Inflammatory reactions are combined with high protein degradation and this signifies an enhanced performance of intracellular transport. Golgi apparatus and endoplasmatic reticulum are in progress and in steadily rebuild. Alleviation ER-stress and therefore ER-specific apoptotic pathways, by AMPK activation, protects cardiomyocytes from a hypoxic injury [25]. In summary, AMPK is involved in cellular apoptosis pathways and mediates autophage activity during myocardial ischemia [22-25].

## AMPK AND CARDIAC REMODELING

Cardiac ventricular hypertrophy is associated with upstream gene expression and subsequent changes in transcription and translation, which result in an increase in cardiac myocyte volume. This increase in volume reflects the changes in response to physical and chemical alterations. Terai *et al*. described an increase of cardiac AMPK’s α1 and α2 - subunits in chronic hypertrophied hearts [25]. LKB 1 (tumor suppressor kinase), a serine/threonine kinase, modulates AMPK to inhibit the m-target of rapamycin (mTOR) signaling. Accordingly, cell growth and proliferation are each down regulated and suppressed [3]. mTOR signaling is involved in different parts of intracellular signal transduction e.g. gene transcription and gene translation or a central regulation point of cell proliferation.

Various cell types, e.g. cardiomyocytes and extracellular matrix (ECM), are active in proliferation and differentiation as an adaption to changes in physiological conditions. Specifically, AMPK plays a central role in myocardial remodeling by attenuating the growth and proliferation of cardiac fibroblasts. The extracellular signal-regulated kinase (ERK), which influences the growth and proliferation of cardiac fibroblasts, exhibits an interaction with AMPK [26-28]. 

The role of AMPK during periods of myocardial stress or cardiac hypertrophy, e.g. aortic constriction or hypertension, is highly controversial [4, 5, 11, 29-32]. The exact steps necessary for AMPK-regulated physiological hypertrophy is currently widely debated. However, without the diverse regulation of various targets by AMPK, cell death and ultimately necrosis as seen in multiple cases of heart disease, occurs [5, 29-31, 33].

## OUTLOOK

AMPK, with its multiple functional variations in the energy metabolism of the heart, owns a huge potential to treat cardiovascular diseases in several different ways [33]: e.g. it may reduce post-ischemic injury, can support surgical interventions or is even able to degrade risk factors, which lead to hemodynamic disorders [27, 34-36]. Therefore, in the future, AMPK may play a key role to approach metabolic dysfunctions and will consequently improve the therapy of heart failure [36, 37].

## SUMMARY

AMPK is an important regulatory enzyme during periods of myocardial stress, regulating energy metabolism, protein synthesis and cardiovascular protection. Once activated by the depletion of ATP, AMPK reversibly phosphorylates and dephosphorylates key enzymes in the heart’s energy metabolism. 

Thus, AMPK plays a key role in the coordination of the heart’s anabolic and catabolic pathways.
